# The influence of physical exercise on attentional bias: a systematic review and meta-analysis

**DOI:** 10.3389/fpsyt.2026.1889943

**Published:** 2026-07-28

**Authors:** Chuan Song, Zhifeng Wang, Shuolong Li, Haihong Xue, Xingtong Wang, Dongmei Wang

**Affiliations:** 1 College of Rehabilitation Engineering, China Civil Affairs University, Beijing, China; 2 Department of Physical Education, Xi’an Polytechnic University, Xi’an, Shanxi, China; 3 School of Public Health, Shandong First Medical University & Shandong Academy of Medical Sciences, Jinan, Shandong, China; 4 Department of General Education, Shandong First Medical University & Shandong Academy of Medical Sciences, Tai’an, Shandong, China; 5 College of Sports Medicine and Rehabilitation, Shandong First Medical University & Shandong Academy of Medical Sciences, Tai’an, Shandong, China

**Keywords:** attentional bias, behavioral disorder, physical activity, physical exercise, psychological disorder

## Abstract

**Background:**

Attentional bias refers to the tendency to prioritize attention toward negative or threatening stimuli rather than positive or neutral ones. It is closely linked to a range of psychological and behavioral disorders. Physical exercise, a behavior beneficial to both physical and mental health, has been shown to influence attentional bias. This study aims to systematically review the literature and conduct a meta-analysis on the relationship between physical exercise and attentional bias, providing a comprehensive overview of current research in this field.

**Objective:**

To investigate the relationship between physical exercise and attentional bias across different populations, thereby providing insights for interventions targeting psychological distress and maladaptive behaviors.

**Methods:**

Literature was retrieved from eight databases, followed by study screening, data extraction and analysis. A qualitative synthesis was conducted, while a quantitative analysis of relevant variables was performed using meta-analysis.

**Results:**

A total of 32 studies were included in this review. Physical exercise was found to reduce negative attentional bias in individuals with anxiety, depression, and high psychological stress, alleviating symptoms of these conditions. However, physical exercise did not significantly reduce negative attentional bias in obese or overweight individuals. It also reduced attentional bias towards addiction-related cues in addicted individuals and alleviated craving for addictive substances or behaviors. Furthermore, it reduces negative attentional bias and enhances positive attentional bias in healthy individuals, thereby promoting healthy behaviors. The effect of physical exercise on negative attentional bias appears to be modulated by exercise intensity, with moderate-intensity exercise demonstrating superior efficacy. In contrast, acute high-intensity exercise can increase an individual’s negative attentional bias.

**Conclusion:**

Moderate-intensity physical exercise appears effective in reducing negative attentional bias and enhancing positive attentional bias, particularly when assessed using emotionally salient image stimuli. However, effects may be attenuated or reversed under conditions of high-intensity exercise, abstract verbal stimuli, or in specific populations such as obese individuals undergoing high-energy expenditure. Therefore, moderate-intensity exercise may serve as an effective intervention for improving psychological and behavioral disorders.

## 1 Introduction

Attentional bias refers to the tendency of individuals to prioritize attention toward negative or threatening stimuli over neutral ones, thereby rendering them more sensitive to such information (1). For example, individuals with addiction can rapidly detect addiction-related stimuli among numerous neutral stimuli and focus preferentially on this information. This bias towards negative or threatening stimuli enables individuals to respond to potential threats quickly and effectively, holding evolutionary significance for survival. However, excessive attentional processing bias can lead to maladaptation to the environment and cause serious psychological or behavioral disorders (2). For instance, numerous studies have established a link between anxiety and attentional bias towards threatening stimuli (3, 4). A meta-analysis involving 172 studies further revealed that various anxiety groups (clinical, non-clinical, children and adults) exhibited attentional bias towards negative or threatening stimuli across different experimental paradigms and conditions (5). Conversely, such an attentional bias was not observed in non-anxious individuals. Accordingly, the cognitive theory of anxiety proposes that attentional bias towards threatening stimuli is the core mechanism underlying the development and maintenance of anxiety symptoms and disorders (6, 7). The severity of attentional bias is positively correlated with the severity of anxiety, and vice versa (8). Aktar et al. (9) also reported that parents’ anxiety levels can prospectively predict children’s visual search attentional bias, suggesting that attentional bias may be an important mechanism for the intergenerational transmission of anxiety. Research on depressed individuals has also found that they exhibit a significant attentional bias towards negative emotional cues in facial expressions (10). Everaert et al. (11) and Gotlib et al. (12) further found that, compared with healthy controls, depressed individuals exhibit increased attentional bias towards negative information and decreased bias towards positive information. Stein et al. (13) also reported that mothers with postpartum depression exhibit attentional bias towards the negative emotional faces of their infants. Todd et al. (14) further found in a meta-analysis that attentional bias variability was positively correlated with the severity of trauma-related symptoms (r = 0.21–0.25) and was also associated with anxiety and depression. Other studies have demonstrated that individuals with eating disorders have a higher attentional bias towards food and body-related stimuli than healthy controls (15). Furthermore, research on heroin (16), mobile phone (17), and alcohol addiction (18), as well as smoking (19), indicates that attentional bias towards addictive cues is a critical factor in the development, maintenance or relapse of addictive behaviors. Clinically, interventions that reduce negative attentional bias can effectively alleviate psychological and behavioral disorders. For example, training individuals to direct attention away from threatening stimuli can alleviate social phobia (20, 21) and generalized anxiety disorder (22), whereas training attention towards such stimuli exacerbates anxiety (23). Consequently, some researchers regard the reductions of negative attentional bias as a key metric for evaluating the efficacy of interventions targeting adverse mood and behavioral disorders (24, 25). Interventions that correct attentional bias hold substantial theoretical and practical value for the treatment of psychological disorders and the modification of maladaptive behaviors.

Currently, intervention research on attentional bias primarily focuses on cognitive psychology and pharmacology. Attentional bias modification (ABM) is a widely recognized cognitive method that effectively modifies attentional bias (26). ABM directly modifies attentional bias toward emotional information through a bottom-up strategy, primarily using implicit training to reduce conscious reactivity to negative stimuli (such as hypervigilance or difficulty in attention dissociation), thereby altering negative attentional bias. Research on patients with anxiety has demonstrated ABM’s efficacy in reducing negative attentional bias (27). However, ABM typically requires professional experimental paradigms, such as dot-probe tasks. In these tasks, participants must respond to neutral or positive stimuli while ignoring threatening ones, or select target images from a series of distractors (e.g., selecting positive faces from neutral ones). The complexity of these procedures, along with the need for specialized materials and trained personnel, hinders their widespread implementation among the general public. Furthermore, some researchers have questioned the efficacy of ABM, arguing that it fails to alleviate social anxiety symptoms at clinical and subclinical levels (28), thereby implying its limitations as a standalone treatment. Pharmacological studies have confirmed that selective serotonin reuptake inhibitors (SSRIs) exert anti-anxiety effects by improving anxiety-related emotional processing biases (29). SSRIs not only reduce anxiety but also decrease vigilance towards threatening stimuli (30). Stein et al. (13) also found that both the antidepressant serotonin norepinephrine reuptake inhibitors (SNRIs) and SSRIs can enhance the attentional bias of postpartum depressed mothers towards their infants’ positive emotional faces and alleviate postpartum negative emotions by changing the evaluation of infants’ facial expressions. Although pharmacological methods are valuable for reducing negative attentional bias and treating psychological disorders, long-term pharmacotherapy is associated with side effects and a significant economic burden. Therefore, There is an urgent need to explore novel, accessible, scalable, and cost-effective intervention strategies that can reduce negative attentional bias and thereby ameliorate psychological or behavioral disorders.

Physical exercise exerts positive intervention effects on various psychological disorders and addictive behaviors. It plays a unique role in maintaining physical and mental health, ameliorating mental disorders, and alleviating psychological stress (31–33). Compared with psychotherapy and pharmacotherapy, physical activity offers significant advantages, including reduced stigma, minimal side effects, and high accessibility (34). Physical exercise can modulate physiological and emotional processing in a bottom-up manner (35). Recent studies indicate that physical exercise not only reduces negative attentional bias, but also accelerates attention dissociation from negative emotional information or threatening stimuli (36, 37). In 2020, the World Health Organization (WHO) issued the “Guidelines on Physical Activity and Sedentary Behavior”, which detailed physical activity recommendations for children, adolescents, adults, older adults, pregnant women and individuals with chronic conditions. These guidelines provide an important reference and basis for exercise interventions targeting negative attentional bias across diverse populations, thereby further stimulating research interest in this field.

To date, multiple meta-analyses and systematic reviews have examined attentional bias in individuals with conditions such as obesity (38), anxiety (5), depression (39), post-traumatic stress disorder (14), phobic disorders (40), and substance addictions including nicotine (41) and alcohol (42). However, no systematic review has specifically examined the relationship between physical exercise and attentional bias. This gap hinders a holistic understanding of the field and impedes the formulation of exercise-based intervention strategies for negative attentional bias. To address this, the present study aims to systematically review the existing literature on the effects of physical exercise on attentional bias, integrating perspectives from exercise science and cognitive psychology to characterize the current state of research. Focusing on populations with depression, anxiety, obesity, and addiction, this review seeks to offer valuable insights for improving maladaptive psychological states and behaviors in these groups.

## 2 Methods

### 2.1 Research question formulation

Given the novelty of this field and the heterogeneity of existing literature, a broad approach was adopted to cover a wide range of studies and topics. Initially, an exploratory literature search we conducted. Following screening, inclusion and exclusion criteria were defined iteratively to address three core research questions: (a) Which populations are susceptible to attentional bias, and what is the relationship between attentional bias and psychological outcomes or adverse behaviors? (b) How does physical exercise intervention affect attentional bias under different conditions? (c) What are the physiological or psychological mechanisms underlying the effects of physical exercise on negative attentional bias?

### 2.2 Data sources and retrieval strategies

After defining the research question, a comprehensive literature search was conducted using the following strategy. The following keywords were used: “attentional bias”, “dot-probe task”, “dot-probe protocol”, “attention”, “attention bias modification” or “cognitive bias”, “emotion”, “Stroop test”, “physical exercise”, “physical activity”, “exercise intervention” and “exercise” to search for English and Chinese literature in China National Knowledge Infrastructure, WanFang Data, PubMed, SpringerLink, ScienceDirect, PsycINFO, Scopus and Web of Science from database inception to December 31, 2025.

The search strategy was defined as follows: (“attentional bias” OR “attention bias modification” OR “cognitive bias” OR “dot-probe task” OR “dot-probe protocol” OR “attention” or “Stroop test”) and (“physical exercise” OR “physical activity” OR “exercise intervention” OR “exercise”).

### 2.3 Eligibility criteria

Inclusion criteria: (1) Peer-reviewed journal articles published in Chinese or English in the aforementioned databases; (2) Studies investigating attentional bias (including both positive and negative biases) in diverse populations, such as individuals with psychological disorders, substance addiction, behavioral addiction, or negative emotional states; (3) Availability of full text (including academic papers, theses, and conference proceedings); (4) Quantitative and qualitative research papers exploring the relationship between physical exercise/physical activity/exercise intervention and attentional bias; (5)To comprehensively assess the relationship between physical exercise and attentional bias, observational studies or experiments utilizing exercise-related words or images as stimuli (without an exercise intervention) were also included.

Exclusion criteria: Studies were excluded if they met any of the following criteria: (1) Duplicate publications; (2) Studies without accessible full text (e.g., conference abstracts); (3) Significant deviations from the intended intervention protocols, missing outcome data, or vague/unclear conclusions.

### 2.4 Data extraction

For the included studies, the following information was extracted: study design, participant characteristics, experimental paradigm, experimental materials, and key findings.

### 2.5 Data synthesis

A narrative synthesis was conducted to analyze the results of all included papers. Additionally, descriptive statistics were used to summarize the characteristics of the studies (see **Table 1**).

**Table 1:** A schedule of studies on the effect of physical exercise on attentional bias.

Author	Country	Study design	Participants	Sample number	Experimental paradigm	Materials and duration	Physical exercise program	Assessment tools/equipment	Main study findings
Barnes et al. (44)	America	Balanced crossover design	People with trait anxiety (age:19.8 ± 0.9)	30 (male:19; female:11)	Dot-probe task	Neutral-positive and neutral-negative image pairs (presentation time:500ms)	A 30-minute moderate-intensity cycling (70% HRR)	Physical activity readiness questionnaire, state-trait anxiety scale, positive and negative mood scale, modified Baker depression scale	Physical exercise reduced attentional bias toward negative cues and increased attentional bias toward positive cues in anxious individuals. Following the exercise intervention, positive emotions increased significantly, while negative emotions and anxiety levels decreased.
Berry (45)	Canada	Group control	Undergraduate (age:20.01 ± 1.93)	72 (male:28; female:42; unclear:2)	Stroop task	Movement- control word pairs and sedentary-control word pairs (presentation time:2000ms)	No physical exercise intervention	Physical exercise Schema Questionnaire	Regular exercisers demonstrated an attentional bias toward exercise-related stimuli, whereas infrequent exercisers showed a bias toward sedentary-related stimuli.
Berry et al. (46)	Canada	Cross-sectional survey	College students (age:19.45 ± 1.76)	72 (male:50; female:22)	Visual dot-probe task	Exercise-neutral image pairs (presentation time:2000ms)	No intervention	The Godin Leisure-Time Exercise Questionnaire, Explicit Attention Questionnaire	More active participants exhibited an attentional bias toward exercise-related images. Furthermore, attentional processing of physical activity stimuli may vary by gender. For men, regardless of their usual activity level, all exhibited an attentional bias toward exercise-related images. In contrast, for women, only the most active individuals showed a bias toward physical activity, while the least active showed a bias toward neutral (control) images.
Brunyé et al. (47)	America	Balanced crossover design	Healthy adults (age:19.8)	12 (male:12; female:0)	Eye tracking technology	Threatening (included weapon)-neutral image pairs (presentation time:3000ms)	A 30-minute low-intensity (25%-35% HRmax) or high-intensity (75%-85% HRmax) cycling.	—	High-intensity training increased individual sensitivity to details of scenes containing weapons (threatening stimuli) by approximately 27%. Consequently, high-intensity physical exercise induced an attentional bias toward threatening scene details in individuals.
Calitri et al. (48)	England	Cross-sectional survey	College students (age:23 ± 6)	125 (male:90; female:34; unclear:1)	Visual dot-probe task and Extrinsic affect Simon task (EAST)	Exercise- non-exercise word pairs (presentation time:500ms)	No intervention	Structural interview method: 7-day physical activity recall (PAR)	Self-reported physical activity over the past seven days was positively correlated with individual attentional bias toward exercise-related words. Implicit attitude was positively correlated with previous physical activity; furthermore, more extreme implicit attitudes (whether positive or negative) were associated with greater attentional bias toward exercise-related cues. Explicit attitude (affective and instrumental) moderated the relationship between physical activity and attentional bias.
Cheval et al. (49)	Switzerland	Group control	College students (age:21.7 ± 3.8)	77 (male:25; female:52)	Visual dot-probe task and Eye tracking technology	Five pairs of images related to physical activity and inactivity (presentation time: 4000-4500ms)	No intervention	International Physical Activity Questionnaire (IPAQ)	Compared with less active participants, more active participants exhibited a greater attentional bias toward physical activity stimuli.
Clarke et al. (50)	England	Randomized control	Experiment 1 Healthy college students (age: 21-40) Experiment 2 Mass fitness enthusiast (age:20-47)	Experiment 1 24 (male:6; female:18) Experiment 2 48 (male:19; female:29)	Scrambled sentence test (SST)	Interpret twenty sentences in four minutes	Experiment 1: A brisk walk lasting 30 minutes Experiment 2: A treadmill physical exercise lasting 20–30 minutes (85% HRR)	State-Trait Anxiety Inventory (STAI); Beck Depression Rating Inventory (BDI-II); Stress Perception Scale (PSS)	Experiment 1: Participants’ trait anxiety levels decreased after walking, but no significant change was observed in negative interpretational bias. Experiment 2: Negative interpretational bias decreased following moderate-intensity physical exercise, alongside reductions in anxiety, depression and stress levels.
Cooper et al. (51)	America	Randomized module design	The anxious (age:18-34)	Experiment 1 64 (male:0; female:64)( Experiment 2 63 (male:0; female:63)	Dot-probe task	Experiment 1: Neutral-neutral and emotional-neutral word pairs (presentation time:500ms) Experiment 2: Neutral-neutral and threatening- neutral image pairs (presentation time:500ms)	One 20-minute moderate-intensity cycling (45% VO_2_R)	Beck Depression Rating Inventory (BDI-II) and State Trait Anxiety Scale (STAI-T)	Experiment 1: Physical exercise did not reduce attentional bias toward textual information in anxious individuals; Experiment 2: Physical exercise reduced attentional bias toward threatening images in anxious individuals. Furthermore, it enhanced the efficiency of attention dissociation from threatening stimuli and shifting attention toward neutral stimuli.
Cui et al. (74)	China	Randomized control	College students with high psychological stress levels (aged: 19.24 ± 1.18)	42 (male:11; female:31)	Word−face Stroop task, Memory bias task, Interpretation bias task	Emotion face-emotion word (presentation time:200ms), Memory bias task (presentation time:2000ms), Interpretation bias task (presentation time:5000ms)	A single 30-minute session of basketball exercise (60-70% of maximum oxygen)	The Chinese version of the Perceived Stress Scale (CPSS)	1.Attentional bias test: The exercise group exhibited faster responses to positive emotional stimuli and slower responses to negative stimuli. 2.Memory bias test: The exercise group demonstrated a significantly lower error rate for negative emotional faces compared to the non-exercise group. Specifically, the non-exercise group showed a significantly higher error rate for negative faces compared to neutral faces, whereas no significant difference was observed in the exercise group. 3.Interpretation bias test: The non-exercise group exhibited a negative interpretative bias, whereas the exercise group showed reduced negative interpretation bias.
Flack et al. (52)	America	Balanced crossover design	Overweight or obese people (age:32.9 ± 7.6)	30 (male:16; female:14)	Dot-probe task and food-related go/no-go task	Food-neutral image pairs and neutral-neutral image pairs (presentation time:3000ms)	Engaging in a 500-kilocalorie- burning physical exercise on the fitness meter (with the intensity of own choice)	Visual analogue scale (VAS)	An acute bout of physical exercise increased attentional bias toward food cues in obese/overweight individuals, while inhibitory control did not change significantly. No significant correlation was found between attentional bias and inhibitory control. Furthermore, neither attentional bias nor inhibitory control was associated with hunger levels.
Giel et al. (53)	Germany	Group control	15 patients with anorexia nervosa (AN) (age:23.9± 4.9) 15 healthy endurance athletes (age:24.5 ± 3.0) 15 non-athletes (age:24.7 ± 2.8)	45 (male:0; female:45)	Eye tracking technology	Physical active- non-physical active image pairs (presentation time:3000ms)	No intervention	International Physical Activity Questionnaire ((IPAQ-short form), Behavioral activation score (BAS), Behavioral inhibition score (BIS), State-Trait Anxiety Inventory (STAI), Patient Health Questionnaire (PHQ), Eating Disorder Inventory (EDI-2)	Compared with non-athletes, patients with anorexia nervosa and athletes exhibited higher levels of attentional engagement toward images of physical activities, specifically, an attentional bias. The amount of physical activity was associated with attentional orientation and engagement. Furthermore, compared with non-athletes, both patients with anorexia nervosa and athletes rated physical exercise stimuli as more pleasant.
He et al. (32)	China	Randomized control	Methamphetamine use disorders (MUDs) (age:36.30 ± 8.22)	32 (male:32; female:0)	Drug color-related Stroop task	Drug-related words and neutral words (presentation time:1000ms), randomly presented colors: red, yellow, green and blue.	Tai Chi of 3 times/week, 45 min/session, for 4 weeks.	Visual analogue scale (VAS)	A four-week Tai Chi intervention reduced sensitivity and attentional bias toward drug-related cues, as well as subjective drug craving in patients with MUD.
Heenan et al. (54)	Canada	Two-factor mixed factorial design	Undergraduate and postgraduate (age:18-26)	55 (male:23; female:32)	Perceptual task	Depictions of solid cubes and stick figure walkers (presentation time:500ms)	One 10-minute walking (4 km/h) or jogging (8 km/h)	State-Trait Anxiety Inventory (STAI)	Walking or jogging reduced participants’ attentional bias toward the audience and also decreased their anxiety levels.
Julian et al. (55)	America	Randomized control	Anxious college students (age:19.87 ± 3.15)	112 (male:91; female:21)	Dot-probe task	Neutral- threatening word pairs and neutral-neutral word pairs (presentation time:500ms)	One 20-minite moderate-intensity (65-70% HRmax) running	Depression-Beck Depression Rating Inventory (BDI-II), The Liebowitz Social Anxiety Scale-Self-Report, (LSAS-SR), State-Trait Anxiety Inventory (STAI)	A single session of acute moderate-intensity aerobic exercise did not alter attentional bias toward threatening words in anxious individuals.
Ligeza et al. (56)	Poland	Randomized crossover design	Healthy adults (age:18-35)	24 (male:12; female:12)	mage Perception Tasks	Pleasant scene-unpleasant scene image pairs (presentation time:600ms; stimulation interval: 1000-2000ms)	A 30-minute low-intensity (30%-40% HRR) and moderate-intensity (60%-70% HRR) power cycling or rowing.	Magneto- encephalography	Participants exhibited a negative attentional bias toward unpleasant scene images in a resting state. This negative attentional bias was attenuated following low-intensity physical exercise and was reversed following moderate-intensity physical exercise. Physical exercise decreased the brain’s processing time and intensity for negative scenarios, while increasing the processing time and intensity for positive scenarios.
Liu et al. (58)	China	Randomized control	Methamphetamine dependency (age:18–38)	100 (male:100; female:0)	Modified dot-probe paradigm, 2-back task, Shift task	Neutral- methamphetamine-related negative images (presentation time:500ms)	A total of 8 weeks of treadmill physical exercise, 5 times/week, 60 min/session (60–80% HRmax)	The Self-Anxiety Scale (SAS), The Self-Depression Scale (SDS), Barratt Impulsiveness Scale (BIS-11), The Self-Control Scale (SCS), Visual analogue scale (VAS)	In the physical exercise intervention group, reaction time in the dot-probe task decreased. Furthermore, accuracy in the 2-back task was significantly higher compared to the attentional bias task (ABT) group and the control group. Levels of anxiety and depression were lower than pre-intervention levels, and drug craving was reduced.
Liu et al. (57)	China	Randomized control	Methamphetamine use disorders (MUDs) (aged:19-36)	100 (male:100; female:0)	Dot-probe paradigm, Stroop task, 2-Back task, Shift task	Neutral- methamphetamine-related image pairs (presentation time:500ms)	3 times/week, 60 min/session, for 8 weeks of physical exercise intervention (60%–85% HRmax); Physical exercise combined attentional bias correction training; Attentional bias correction training: 3 times/week, 60 min/session, for a total of 8 weeks; Control group: 3 times/week, 60 min/session, for a total of 8 weeks of health education.	The Self-Rating Anxiety Scale, the Self-Rating Depression Scale, the Self-Control Scale, a visual analog scale	In the physical exercise intervention group, reaction time in the dot-probe task decreased, although attentional bias did not change significantly. Executive function, heart rate variability (HRV), and cardiopulmonary fitness were significantly improved in both the combined intervention group and the aerobic exercise group. Additionally, high-frequency HRV and cardiopulmonary fitness levels increased significantly in the aerobic exercise group, alongside a reduction in drug craving.
Marotta et al. (59)	Italy	Before and after comparison	Health volunteers (age:21-65)	46 (male:33; female:13)	Dot-probe task	Emotions (happy, anger, sad) -neutral and neutral-emotional face pairs (presentation time:1000ms)	21km running race (half marathon) (duration> 1h)	State-Trait Anxiety Scale Y1 (STAIY1), Positive and Negative Affect Scale (PANAS)	Following physical exercise, young individuals exhibited an attentional bias toward anger and attention dissociation from sadness, whereas older adults showed an avoidance bias toward anger. Thus, attentional bias was modulated by age. The regression lines for angry and sad expressions intersected at approximately 45 years of age. Additionally, anxiety levels decreased significantly after running.
Oh et al. (60)	England	Balanced crossover design	Overweight and normal weight fasting people (age:18-45)	58 (male:0; female:58)	Dot-probe task	Neutral-chocolate image pairs (presentation time:200ms (capture IAB) or 1000ms (capture MAB))	One 15-minute moderate-intensity cycling (RPE: 11-13)	0–100 visual analogue score	Physical exercise reduced participants’ initial attentional bias toward chocolate images. Regardless of body mass index (BMI) or fasting status, physical exercise reduced both chocolate craving and attentional bias toward chocolate images.
Oh et al. (61)	England	Randomized crossover design	Those who quit snacking and smokers (age:23.96 ± 4.83)	23 (male:15; female:8)	Eye tracking technology	Snacks-neutral or smoking-neutral image pairs (presentation time:8200ms)	One 15-minute session of moderate-intensity (40%–50% HRR, RPE: 11-13) and high-intensity (70%–75% HRR, RPE: 15-17) cycling	Smoking Desire Intensity and Food Craving Questionnaire - National Version (FCQ-S)	Both moderate-intensity and high-intensity physical exercise reduced participants’ initial attentional bias (IAB) toward cigarettes and snacks. However, only high-intensity exercise reduced maintenance attentional bias (MAB). Furthermore, physical exercise reduced participants’ desire to smoke and craving for snacks.
Tao et al. (64)	China	Randomized control	College students with high psychological stress (age:19.12 ± 1.02)	47 (male:23; female:24)	Emotional Stroop task	Positive, neutral and negative images, nine each (presentation time: not specified)	3 times/week, 30 min/session, for 12 weeks of aerobic physical exercise (mean HR: 148.878 ± 10.372 bpm)	“Psychological Stress Scale for Chinese College Students”, ERP technology	Following a physical exercise intervention, attentional bias toward negative emotions decreased in college students with high psychological stress, and the time required to disengage from negative emotions was reduced. Thus, physical exercise enhanced the emotional attention function of these students.
Taylor et al. (63)	England	Randomized control	Smoker (age: 30.1 ± 9.7)	60 (male:26; female:34)	Stroop task	Cigarette-related words (presentation time:1000ms)	A 15-minute moderate-intensity treadmill physical exercise	Cigarette craving Questionnaire, Mood and Physical Symptoms Scale, test for withdrawal symptoms	Physical exercise reduced participants’ attentional bias and craving toward cigarettes, and alleviated withdrawal symptoms induced by cigarette cues. Following exercise, craving for smoking, tension, stress and inattention did not increase upon exposure to smoking cues.
Taylor et al. (62)	England	Balanced crossover design	Abstainer (age:20.8 ± 0.8)	20 (male:9; female:11)	Dot-probe task	Neutral-alcohol image pairs (presentation time:200ms (capture IAB) or 1000ms (capture MAB))	One 15-minute treadmill run (average HR:124.80 ± 11.55, RPE: 11-13)	Alcohol Impulse Questionnaire (AUQ) and Self-Efficacy Questionnaire for Refusing to Drink-Revised	Physical exercise reduced participants’ maintenance attentional bias (MAB) toward alcohol cues, as well as alcohol craving and consumption.
Tian et al. (66)	America	Balanced order	Healthy college students (age:18-35)	34 (male:17; female:17)	Dot-probe task	Emotional (pleasant or unpleasant)- neutral face pairs (presentation time:1000ms)	One moderate-intensity (45% VO_2_peak) and high-intensity cycling (80% VO_2_peak)	State-Trait Anxiety Inventory (STAI)	After moderate-intensity physical exercise, participants’ attentional bias toward pleasant faces increased, whereas bias toward unpleasant faces decreased. No significant differences in attentional bias scores for emotional faces were observed between the resting state and high-intensity physical exercise. These findings suggest that during moderate-intensity physical exercise, attentional resources shift from unpleasant emotional stimuli to pleasant ones.
Van Rensburg et al. (33)	England	Randomized crossover design	Smoker (age:20-50)	20 (male:15; female:5)	Eye tracking technology	Smoking-neutral image pairs (presentation time:1000ms)	One 15-minute moderate-intensity cycling (49.5% HRR), RPE: 11–13)	Seven-point Subjective Cravings scale	Physical exercise reduced participants’ total fixation duration on smoking images and their initial attentional bias toward such images. It also reduced the desire for cigarettes.
Wang et al. (67)	China	Randomized control	Anxious college students (age:20.11 ± 1.21)	50 (male:23; female:27)	Dot-probe task	Negative-negative face pairs and neutral-neutral face pairs (presentation time:500ms)	A total of 12 weeks of aerobic physical exercise intervention, 3 times/week, 60 min/session (60%–80% HRmax)	The Self-Anxiety Scale (SAS), Physical activity Rating Scale (PARS-3)	Physical exercise reduced attentional bias and dissociation difficulty from negative emotional faces in anxious individuals, but had no significant effect on attentional orientation. Additionally, physical exercise reduced participants’ anxiety levels.
Yang et al. (68)	China	Randomized balanced order	Depressed individuals (age:19.96± 0.83) Non-depressed individuals (age:19.66 ± 0.92)	47 (male:39; female:9) 47 (male:37; female:10)	Spatial cue paradigm	Emotional images (positive or negative) (presentation time:200ms)	A 30-minute aerobic cycling (60%-70% HRmax)	State-Trait Anxiety Inventory (STAI), and Trait Anxiety subscales of the Beck Depression Rating Inventory (BDI)-II	A multimodal intervention combining aerobic exercise and rhythmic music enhanced attentional bias toward positive emotions in individuals with mild depression. Conversely, a unimodal aerobic exercise intervention increased attentional bias toward positive emotional cues in non-depressed individuals. For individuals with mild depressive symptoms, the multimodal intervention was more effective than the unimodal intervention, whereas for those without depressive symptoms, the unimodal aerobic exercise intervention proved more effective.
Yue Heng (69)	China	Randomized control	Experiment 1 Internet addicts (age:19.75 ± 1.56) Experiment 2 Internet addicts (age:19.27 ± 1.42) Experiment 3 Internet addicts (age:19.80 ± 1.53)	Experiment 1 75 (male:34; female:41) Experiment 2 74 (male:37; female:37) Experiment 3 76 (male:36; female:40)	Experiment 1 Cue-target task Experiment 2 Learning-recognition task Experiment 3 Word association task	Experiment 1 Neutral-negative face pairs (presentation time:1000ms) Experiment 2: Negative-positive word pairs (presentation time:3000ms) Fuzzy situational sentences (presentation time:5000ms)	A 30-minute low-intensity (50-59% HRmax), moderate-intensity (60-69% HRmax) and high-intensity (70-79% HRmax) power cycling.	Experiment 1: Bergen Social Media Addiction Scale (BSMAS), The Brief Assessment of Mood (BAM). Experiment 2: BSMAS, Depression-Anxiety-Stress Scale (DASS-21); Experiment 3: Same as Experiment 2	Experiment 1: Both low-intensity and moderate-intensity physical exercise significantly reduced attentional bias toward negative faces in individuals with internet addiction, whereas high-intensity physical exercise did not. Additionally, low- and moderate-intensity physical exercise improved negative mood in these individuals. Experiment 2: Following moderate- and low-intensity physical exercise, participants showed increased retention of positive words, and decreased retention of negative words. In contrast, high-intensity physical exercise had no significant effect on the retention of words of different valences. Experiment 3: Moderate-intensity physical exercise significantly reduced negative interpretational bias and increased positive interpretational bias in participants.
Yun et al. (70)	Canada	Cross-sectional survey	Community residents (age:40.53 ± 13.26)	127 (male:29; female:87; unclear:11)	Visual dot-probe task and Go/No-go task	UWALK-related and healthy U-related image pairs (presentation time:500ms)	No intervention	Seven point bipolar adjective scales, the Godin Leisure-Time Exercise Questionnaire	Attentional bias toward exercise-related images significantly predicted individuals’ engagement in amateur physical activity.
Zhang et al. (71)	Taiwan, China	Random balanced crossover design	Obese adolescents (age:14.63 ± 0.69)	38 (male:18; female:20)	Food cue-related Stroop task	Food and neutral words (presentation time:1000ms), randomly presented color: red, green and blue.	One session of acute moderate-intensity physical exercise (skipping rope) lasting 20 minutes (HR:140-150bpm)	Hunger scale	Following acute physical exercise, obese adolescents exhibited shortened reaction times (RTs) to food-related stimuli, improved inhibitory control, and reduced attentional bias towards food cues.
Zhao et al. (72)	China	Randomized control	Methamphetamine use disorder (age: 29.38 ± 0.56)	61 (male:61; female:0)	Dot-probe task, EEG recording	Drug-related images and neutral image pairs (presentation time:1000ms)	A total of 12 weeks of cycling physical exercise, 3 times/week, 40 min/session. Moderate-intensity physical exercise group (65%–70% HRmax), high-intensity physical exercise group (80%–85% HRmax).	—	Compared to baseline pre-exercise measurements, attentional bias indices were significantly reduced in both the high-intensity and moderate-intensity physical exercise groups post-exercise.
Zhao et al. (73)	China	Randomized control	Women with methamphetamine addiction (age:31.59 ± 2.32)	63 (male:0; female:63)	Dot-probe task	Drug-neutral image pairs (presentation time:1000ms)	3 times/week, 40 min/session, a total of 12 weeks of dance or cycling (65%-70% HRmax)	Electroencephalogram recording	Both dance and cycling improved participants’ early recognition of drug-related cues, weakened drug-related memory, and enhanced attentional control, while also reducing the maintenance of attentional bias toward such cues. Additionally, dance enhanced participants’ emotional control.

### 2.6 Quality assessment

The quality of the included studies was independently assessed by two researchers experienced in cognitive psychology and exercise psychology. A structured tool developed by Brooks et al. (43) was used for the objective assessment, covering research design, data collection and methodology, and the analysis and interpretation of results (presented in the Appendix).

### 2.7 Data transformation and effect size calculation

Two types of comparisons were identified among the included studies. The first involved post-test comparison between groups (exercise group vs. control group), where pre-test data were absent. The second involved pre-post change comparisons, wherein both groups had pre-test and post-test data. In this scenario, change scores were calculated for each group, followed by a comparison of the between-group differences in these changes. For studies lacking pre-intervention data, the Cochrane Handbook for Systematic Reviews of Interventions (Chapter 10, Section 10.12.2) outlines four primary options for handling missing data (1): Analyzing only the available data; (2) Imputing missing data with replacement values; (3) Imputation accounting for uncertainty; and (4) Using statistical models. We adopted the second option, treating pre-test values as a baseline (mean = 0, SD = 0) to calculate between-group differences using change scores. This approach enabled all included studies to be analyzed within a consistent analytical framework. It should be explicitly noted that under the assumption of a zero baseline, the change score mathematically reduces to the post-test score (i.e.,Δ*Me*an=*Me*an *_post_*), and the standard deviation of the change score formula simplifies accordingly. Consequently, for these specific studies, the resulting effect size is mathematically equivalent to a between-group comparison of post-test data. For studies that provided complete pre- and post-test data, the change scores were calculated using the following formula:


$$M_{\text{change}}=M_{\text{post}}-M_{\text{pre}}$$

$$SD_{\text{change}}=\sqrt{SD_{\text{pre}}^{2}+SD_{\text{post}}^{2}-\left(2\times r\times SD_{\text{pre}}\times SD_{\text{post}}\right)}$$

where *M*_post_ and *M*_pre_ represent the post-test and pre-test means for both the exercise and control group, respectively. The standard deviations of the pre-test (*SD*_pre_) and post-test (*SD*_post_) were used to calculate the standard deviation of the change scores. The pre-post correlation coefficient (*r*) was set at 0.6, with sensitivity analyses conducted across a range of 0.6–0.9 (Note that this formula and the *r* assumption apply only to studies with actual pre-test data, as *SD*_pre_ = 0 mathematically nullifies the correlation term in the imputed studies). To assess the robustness of this imputation approach, we conducted an Intraclass Correlation Coefficient (ICC) analysis. Standard deviations derived from comparable studies with consistent designs and paradigms were used to estimate the ICC. Results indicated that ICC values generally ranged between 0.60 and 0.97. While this serves as a robustness check suggesting that the pooled standardized mean difference (SMD) was not unduly influenced by the imputation method, it should not be interpreted as a validation of the zero-baseline assumption.

### 2.8 Meta-analysis

Numerical data from the included studies were extracted using Engauge Digitizer. Meta-analyses were performed, and forest plots were generated, using RStudio.

A variety of methods have been used in the literature to assess attentional bias, such as the dot-probe task, eye-tracking techniques, and the Stroop task. These methods may differ in their measurement scales and numerical ranges. To facilitate the comparative analysis of data obtained using different measurement methods and units, all outcome variables were converted to SMDs for analysis, with 95% confidence intervals (CIs) used for estimation. Heterogeneity was assessed using the *I*² statistic, with I² ≤ 50% indicating low heterogeneity (fixed effects model applied) and I² > 50% indicating substantial heterogeneity (a random-effects model applied). When I² > 50%, subgroup or sensitivity analyses were conducted to explore potential sources of heterogeneity. A *P*-value< 0.05 was considered statistically significant for pooled effect sizes.

## 3 Results

### 3.1 Literature inclusion results

**Figure 1:**
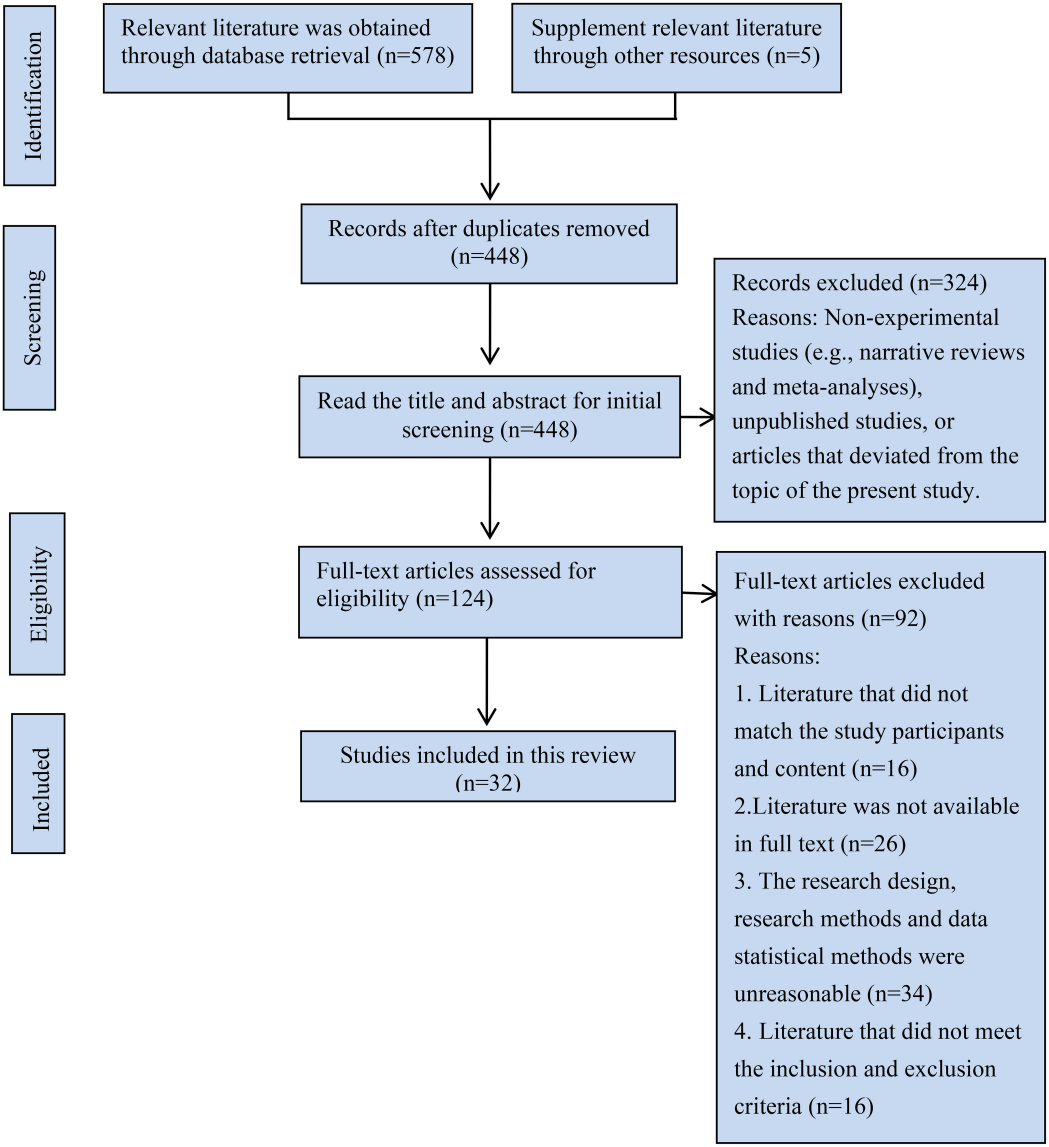
PRISMA flow diagram.

After the initial search, a total of 583 articles were identified from databases and other sources (e.g., WeChat public accounts). Of these, 578 were retrieved from electronic databases including CNKI, WanFang, PubMed, SpringerLink, ScienceDirect, PsycINFO, Scopus, and Web of Science, while five were identified through other sources. After removing 135 duplicates, 324 articles were excluded based on title and abstract screening (214 and 110, respectively). The full texts of the remaining 124 articles were assessed for eligibility, of which 92 were excluded. Ultimately, 32 articles were included in the analysis. A PRISMA diagram illustrating the screening process is presented in **Figure 1**.

From the perspective of publication time, all 32 articles were published after 2000, among which 6 were published between 2000 and 2010, 15 between 2011 and 2020, and 11 after 2020. Geographic Distribution: The studies were conducted by researchers in the United States (n=6), the UK (n=7), China (n=11), Canada (n=4), Germany (n=1), Switzerland (n=1), Italy (n=1), and Poland (n=1). Research Design: The studies employed diverse methodologies, including randomized controlled trials (n=11), crossover studies (n=10), controlled trials (n=3), cross-sectional surveys (n=3), and other experimental designs (n=5). Measurement Tools: The dot-probe task was the most frequently used measure (n=13). Other measures included eye-tracking technology (n=4), the Stroop task (n=6), and various cognitive tasks (e.g., scrambled sentence test, cue-target task). Some studies utilized combined methodologies, such as integrating the dot-probe task with the Go/No-go task (n=2) or eye-tracking (n=1). Most articles were published in English (n=30), with two in Chinese. Study Populations: The participants included individuals with anxiety, depression, internet addiction, and substance addiction, as well as emotionally stimulated individuals and healthy college students. The characteristics of the included studies are summarized in **Table 1**.

### 3.2 The relationship between physical exercise and attentional bias

All 32 studies (32, 33, 44–73) included in this review explored the relationship between physical exercise and attentional bias or interpretational bias, although findings showed notable heterogeneity. Among them, three studies found that physical exercise was associated with increased negative attentional bias (47, 52, 59). Brunyé et al. (47) found that a single session of high-intensity exercise increased attentional bias towards threatening visual scenes. Flack et al. (52) reported that an acute exercise session increased the attentional bias towards food cues in obese or overweight individuals. Marotta et al. (59) also reported that young individuals exhibited increased attentional bias towards angry cues and reduced attention to sad cues following a half marathon, whereas older adults tended to avoid angry information after exercise. Conversely, one study reported no significant effect of physical activity on attentional bias (55). This study involved 112 anxious college students who underwent a 20-minute moderate-intensity running session at 65-70% HR. The results indicated that a single session of acute moderate-intensity aerobic exercise did not alter attentional bias towards threatening words in anxious individuals. The results of the remaining 28 studies (32, 33, 44–46, 48–51, 53, 54, 56–58, 60–73) were largely consistent. Collectively, these studies confirmed from various perspectives that physical exercise may reduce negative attentional and interpretational biases and increase positive attentional or interpretational biases.

### 3.3 The effect of physical exercise on attentional bias in addicted individuals

Ten studies explored the effect of physical exercise on attentional bias in addicted individuals, yielding highly consistent results that indicate physical exercise can reduce attentional bias towards addiction-related cues (32, 33, 57, 58, 61–63, 69, 72, 73). Oh et al. (61) studied individuals with cravings for snacks and smokers, assigning them to a 15-minute session of moderate-intensity (40%-50% HRR, RPE: 11-13) and high-intensity (70%-75% HRR, RPE: 15-17) exercise. They found that participants’ initial attentional bias towards snacks and cigarettes decreased, accompanied by weakened cravings. Similarly, Van Rensburg et al. (33) and Taylor et al. (63) reported consistent results in the populations of smokers. Taylor et al. (62) studied individuals with alcohol dependence and found that after a 15-minute treadmill exercise session (mean HR: 124.80 ± 11.55, RPE: 11-13), attentional bias towards alcohol cues decreased, as did craving for alcohol. He et al. (32), Zhao et al. (73), and Liu et al. (57, 58) examined individuals with methamphetamine use disorder (MUD). They found that, after 4 to12 weeks of moderate-intensity exercise intervention, participants’ sensitivity and attentional bias towards drug-related cues decreased. In 2023, Liu et al. (58) reported that aerobic exercise reduced anxiety, depression, and drug craving in individuals with addiction, while enhancing working memory and cognitive control. In 2024, the same authors (57) further reported that aerobic exercise increased high-frequency heart rate variability (HRV) and improved cardiorespiratory fitness. Zhao et al. (72) compared the effects of a 12-week exercise interventions at moderate (65%–70% HRmax) and high intensity (80%–85% HRmax) on attentional bias in individuals with MUD.

The results showed that attentional bias scores in the dot-probe task were significantly reduced in both exercise groups, whereas no significant changes were observed in the control group. Yue (69) explored the relationship between physical exercise and negative attentional bias in individuals with internet addiction. The study found that low- and moderate-intensity acute exercise significantly reduced attentional bias towards negative faces and negative interpretational bias towards ambiguous situations. Additionally, physical exercise increased individuals’ retention of positive words and enhanced positive interpretational bias towards ambiguous situations.

### 3.4 The effect of physical exercise on attentional bias in individuals with anxiety/depression

Anxiety and depression are common negative psychological states. Among the 32 included studies, five explored the effect of physical exercise on negative attentional bias in individuals with anxiety (44, 51, 55, 67, 68), and one examined its effect on those with depression (68). For example, Barnes et al. (44) recruited 30 individuals with trait anxiety and assigned them to a 30-minute moderate-intensity cycling session (70% HRR). The study found that attentional bias towards negative cues decreased, while attentional bias towards positive cues increased. This shift was accompanied by increased positive emotions and decreased negative emotions and anxiety levels. Wang et al. (67) utilized a dot-probe task to dissect attentional bias into three components: accelerated attentional orientation, difficulty in attention dissociation, and attentional avoidance. Their intervention experiments confirmed that physical exercise reduced negative attentional bias in anxious individuals, primarily by alleviating difficulties in attention dissociation, rather than by altering attentional orientation and avoidance. Furthermore, Wang et al. (67) observed that physical exercise reduced both anxiety levels and negative attentional bias. Yang et al. (68) administered a 30-minute aerobic cycling session (60%-70% HRmax) to both depressed and non-depressed participants. They found that aerobic exercise alone significantly increased attentional bias towards positive cues in non-depressed individuals. However, for individuals with mild depression, a multimodal intervention combining aerobic exercise with rhythmic music was required to significantly increase attentional bias towards positive cues. Inconsistent results were reported by Julian et al. (55), who found that a 20-minute moderate-intensity running session (65-70% HR) did not alter attentional bias towards negative words in anxious individuals. Cooper et al. (51) similarly reported that a 20-minute moderate-intensity cycling session (45% VO_2_R) did not alter attentional bias towards negative textual information in individuals with high or low anxiety, but it did reduce attentional bias towards negative images. The inconsistency in results may be attributable to differences in experimental materials. For textual information, which is relatively abstract and requires semantic coding or threat assessment, the intervention effect of physical exercise may be limited. Consequently, attentional bias towards textual information in anxious individuals did not change significantly following exercise. Conversely, for image cues with high emotional valence and ecological validity (e.g., threatening scenes), the intervention effect of physical exercise was pronounced. Consistent with this, attentional bias towards threatening images was shown to significantly decrease after exercise in individuals with anxiety or depression (44, 67, 68). However, due to the lack of quantitative data regarding processing difficulty or emotional arousal levels across materials, this inference requires further experimental validation.

### 3.5 The effect of physical exercise on attentional bias in obese/overweight individuals

Three studies explored the effect of physical exercise on attentional bias towards food-related cues in individuals with obesity or overweight, yielding inconsistent results (52, 60, 71). Oh et al. (60) reported that a 15-minute moderate-intensity cycling session (RPE: 11-13) reduced initial attentional bias and craving for chocolate images in overweight adults. Zhang et al. (71) reported consistent results when studying obese adolescents. Furthermore, they found that physical exercise not only reduced participants’ attentional bias towards food cues and hunger intensity, but also improved inhibitory control. However, Flack et al. (52) reported contradictory results. They found that a single exercise session expending 500 kcal (at self-selected intensity) increased attentional bias towards food cues in obese individuals, rather than improving their inhibitory control. The inconsistent results among these studies may be related to the difference in energy expenditure during exercise. The exercise protocol used by Flack et al. (52) involved higher energy expenditure (500 kcal), which may have induced a need for rapid energy recovery post-exercise, thereby increasing attentional bias towards food cues. In summary, differences in energy expenditure may be a major contributor to the inconsistent findings across studies.

### 3.6 The effect of physical exercise on attentional bias in healthy individuals

Six studies explored the effect of physical exercise on negative attentional bias in healthy individuals, showing some variation in results (47, 50, 54, 56, 59, 66). Heenan et al. (54) recruited 55 university students and simulated social evaluation scenarios using solid cubes and stick figure walkers. The results showed that after a 10-minute walk, participants’ attentional bias towards the audiences decreased, accompanied by reduced anxiety levels. Ligeza et al. (56) further reported that while healthy adults exhibited attentional bias towards unpleasant scenes at rest, this negative bias weakened after low-intensity exercise and reversed after moderate-intensity exercise. Tian et al. (66) similarly found that after moderate-intensity exercise, healthy college students showed increased attentional bias towards pleasant faces and decreased attentional bias towards unpleasant faces. Clarke et al. (50) used scrambled sentences to investigate the effect of physical exercise on interpretational bias in healthy college students. Results indicated that low-intensity exercise (walking) had no significant effect on negative interpretational bias, whereas moderate-intensity exercise significantly reduced it. However, inconsistent findings were also reported. For example, Brunyé et al. (47) found that high-intensity exercise (75-85% of peak HR) increased attentional bias towards threatening scenes. Marotta et al. (59) also reported that after a half marathon (21 km, duration >1h), younger adults showed increased attentional bias towards angry cues. In contrast, older adults tended to avoid angry cues post-exercise, with an age cutoff point around 45 years. Collectively, these findings suggest that the effect of physical exercise on negative attentional and interpretational bias in healthy individuals is dependent on exercise intensity. Moderate-intensity exercise appears to reduce negative attentional and interpretational bias while increasing positive bias, whereas high-intensity exercise may have the opposite effect.

Five studies explored the effect of physical activity on positive attentional bias in healthy individuals, yielding consistent results (45, 46, 48, 49, 70). Berry (45) used the Exercise Schema Questionnaire to classify 72 healthy college students into three groups: exercise schema, non-exercise schema, and no-schema individuals. Results indicated that non-exercise schema individuals exhibited attentional bias towards sedentary lifestyle-related words, whereas exercise schema individuals showed bias towards exercise-related words. Cheval et al. (49) confirmed that, compared to less active individuals, more active individuals exhibited a greater attentional bias towards exercise-related information. The findings of Berry et al. (46) supported those of Cheval et al. (49), and further suggested that attentional processing of exercise cues may vary by gender. Men exhibited attentional bias towards exercise-related images regardless of their usual activity levels. Conversely, only active women showed attentional bias towards physical activities, inactive women exhibited a bias towards neutral (control) images. Calitri et al. (48) further reported that self-reported physical activity over the past seven days was positively correlated with attentional bias towards exercise-related words. Yun et al. (70) also reported that attentional bias towards exercise-related images significantly predicted levels of leisure-time physical activity. Since both physical activity and exercise are beneficial for individual physical and mental health, attentional bias towards these cues represents a positive cognitive bias. Enhancing this bias suggests that individuals are more likely to engage in physical activity or adopt a more active lifestyle.

### 3.7 The effect of physical exercise on attentional bias in other individuals

Tao et al. (64) investigated the effect of 12-week aerobic exercise intervention (30 minutes per session, three times per week) on attentional bias in college students with high psychological stress. The results showed that the exercise intervention reduced participants’ attentional bias towards negative cues and accelerated recovery from negative emotions. Cui et al. (74) conducted a randomized controlled study involving 42 college students experiencing psychological stress. They found that, following a single 30-minute session of moderate-intensity basketball exercise, the exercise group exhibited lower negative attentional bias and negative interpretation bias compared to the control group. Giel et al. (53) conducted a comparative study involving patients with anorexia nervosa (AN), healthy endurance athletes and non-athletes. They found that, compared to non-athletes, both patients with AN and athletes exhibited a higher level of attentional engagement (e.g., attentional bias) towards exercise images. Furthermore, the amount of physical exercise was associated with attentional orientation and engagement.

### 3.8 Quality of the study

**Figure 2:**
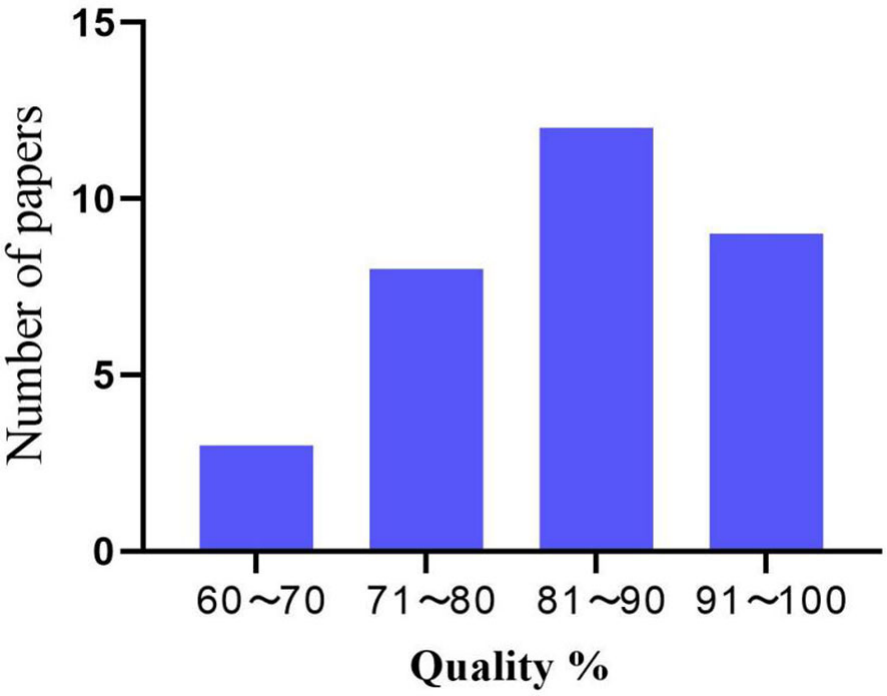
Scores for overall quality.

The quality of the included studies was evaluated using the structured tool developed by Brooks et al. (43). As shown in **Figure 2**, three studies scored between 60% and 70%, eight studies between 71% and 80%, twelve studies between 81% and 90%, and the remaining nine studies scored over 90%. Scores regarding data collection and methodology were mixed, twelve articles received full marks, whereas other studies tended to omit descriptions of participant number at each stage or failed to explain reasons for participant attrition. Sixteen articles scored 100% for the analysis and interpretation of results, while the remainder generally failed to adjust for potential confounding variables or report their data with appropriate caveats. Overall, the studies included in this review demonstrate high quality in terms of research design, data collection, methodology, and the analysis and interpretation of results.

### 3.9 Meta-analysis

As data for relevant variables could not be extracted from two studies (48, 70), a total of 30 studies were included in the meta-analysis.

#### 3.9.1 Meta-analysis of the effect of physical exercise on negative attentional bias in addicted individuals

**Figure 3:**
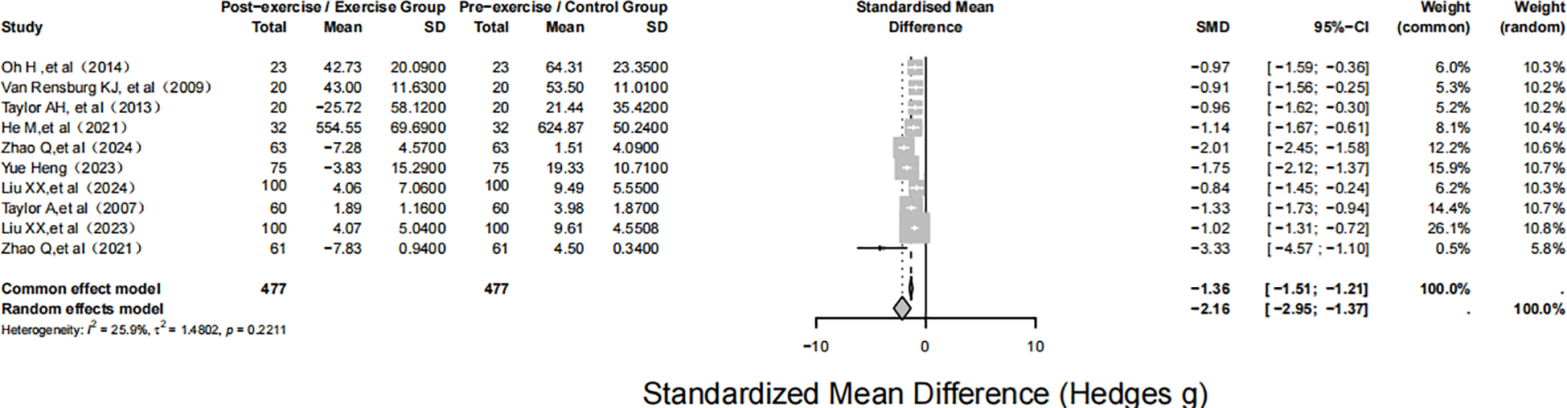
Meta-analysis of the effect of physical exercise on negative attentional bias in addicted individuals [Negative values in the forest plot indicate improvement (reduction in bias), while positive values indicate deterioration (increase in bias). This convention applies to all subsequent forest plots].

Ten studies (32, 33, 57, 58, 61–63, 69, 72, 73) focusing on individuals with addiction utilized image-based materials. The meta-analysis revealed low heterogeneity (I^2^ = 25.9%, *P* = 0.22), therefore, a common-effects model was applied. The results indicated that physical exercise significantly reduced negative attention bias in addicted individuals [SMD=−1.36, 95% CI (−1.51,−1.21), *P* < 0.01]. See **Figure 3**.

#### 3.9.2 Meta-analysis of the effect of physical exercise on negative attentional bias in individuals with anxiety and depression

Five studies (44, 51, 55, 67, 68) involved participants with anxiety and depression, however, one study (68) focused on positive attentional bias and was excluded. Thus, a meta-analysis was conducted on the remaining four studies.

**Figure 4:**
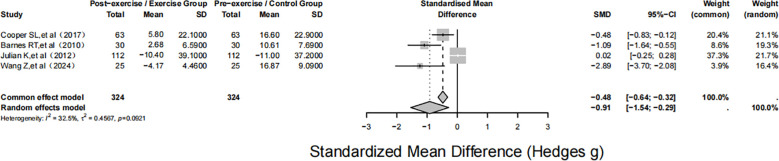
Meta-analysis of the effect of physical exercise on negative attentional bias in individuals with anxiety and depression.

The results showed that heterogeneity among the included studies was low (I^2^ = 32.5%, *P* = 0.09), thus, a common-effects model was applied. The results indicated that physical exercise significantly reduced negative attentional bias in individuals with anxiety and depression [SMD=−0.48, 95% CI (−0.64, −0.32), *P* < 0.01]. See **Figure 4**.

#### 3.9.3 Meta-analysis of the effect of physical exercise on negative attentional bias in overweight/obese individuals

**Figure 5:**
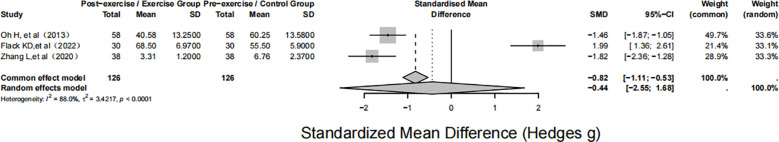
Meta-analysis of the effect of physical exercise on negative attentional bias in overweight/obese individuals.

Three studies (52, 60, 71) investigated overweight or obese individuals using image-based materials. The meta-analysis revealed substantial heterogeneity among the included studies (I² = 88.0%, *P* < 0.01), therefore, a random-effects model was applied. The results indicated that physical exercise did not significantly reduce negative attentional bias in overweight or obese individuals [SMD=−0.44, 95% CI (−2.55, 1.68), *P*> 0.05]. See **Figure 5**.

#### 3.9.4 Meta-analysis of the effect of physical exercise on negative attentional bias in healthy individuals

**Figure 6:**
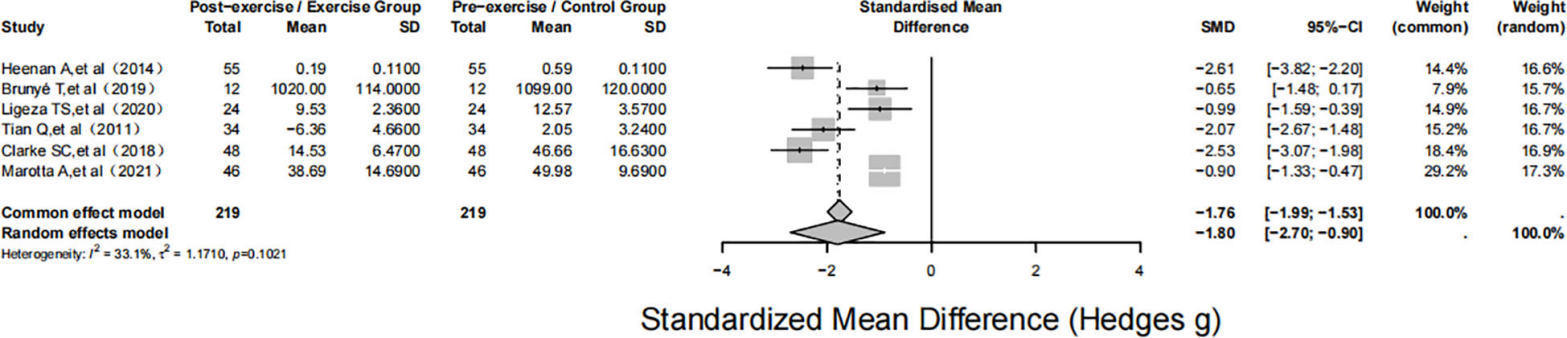
Meta-analysis of the effect of physical exercise on negative attentional bias in healthy individuals.

Six studies (47, 50, 54, 56, 59, 66) investigated the effect of physical exercise on negative attentional bias in healthy individuals. The meta-analysis revealed low heterogeneity among the included studies (I^2^ = 33.1%, *P* = 0.10), therefore, a common-effects model was applied. The results indicated that physical exercise significantly reduced negative attentional bias in healthy individuals [SMD=−1.76, 95% CI (−1.99,−1.53), *P*<0.01]. See **Figure 6**.

#### 3.9.5 Meta-analysis of the effects of physical exercise on negative attentional bias in individuals with high psychological stress

**Figure 7:**
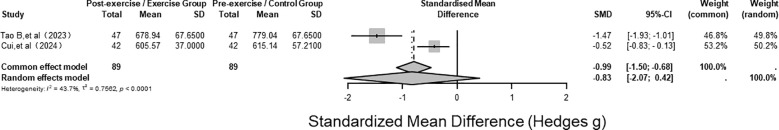
Meta-analysis of the effect of physical exercise on negative attentional bias in individuals with high psychological stress.

Two studies (64, 74) explored the effects of physical exercise on negative attentional bias in individuals with high psychological stress. The meta-analysis revealed low heterogeneity among the included studies (I² = 43.7%, *P<* 0.01), therefore, a fixed-effect model (a common-effects model) was applied. The results indicated that physical exercise significantly reduced negative attentional bias in individuals with high psychological stress [SMD = −0.99, 95% CI (−1.50, −0.68), *P<* 0.01] (see **Figure 7**).

#### 3.9.6 Meta-analysis with word information as experimental materials

**Figure 8:**
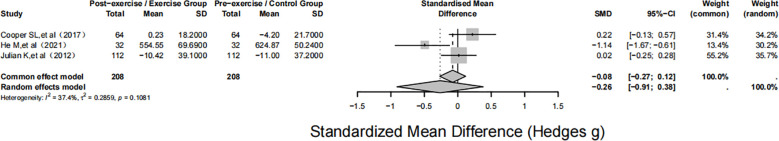
Meta-analysis using word information as experimental materials.

Three studies utilized word-based materials, involving participants with anxiety/depression (51, 55) and individuals with addiction (52). The meta-analysis revealed low heterogeneity among the included studies (I^2^ = 37.4%, *P* = 0.11), thus, a common-effects model was applied. The results indicated that physical exercise did not significantly reduce negative attentional bias [SMD =−0.08, 95% CI (−0.27, 0.12), *P*> 0.05]. See **Figure 8**.

#### 3.9.7 Meta-analysis of the effect of physical exercise on individual positive attentional bias

**Figure 9:**
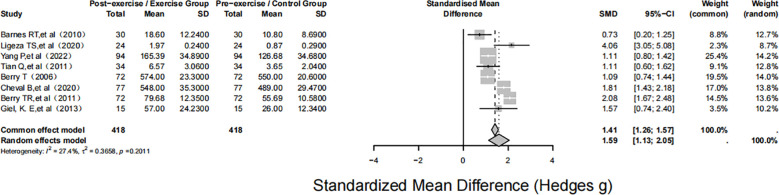
Meta-analysis of the effect of physical exercise on individual positive attentional bias.

Eight studies examined the effect of physical exercise on positive attentional bias (44–46, 49, 53, 56, 66, 68). The meta-analysis revealed low heterogeneity among the included studies (I^2^ = 27.4%, *P* = 0.20), therefore, a common-effects model was applied. The results demonstrated that physical exercise significantly enhanced attentional bias toward positive information [SMD = 1.41, 95%CI (1.26, 1.57), *P* < 0.01]. See **Figure 9**.

#### 3.9.8 Meta-analysis of the effects of different physical exercise intensities on individual negative attentional bias

**Figure 10:**
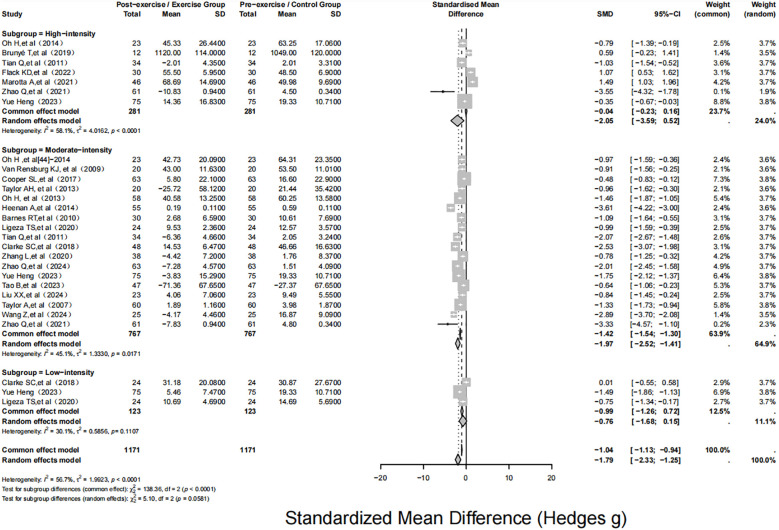
Meta-analysis of the effects of different physical exercise intensities on individual negative attentional bias.

Among the 30 studies included in the meta-analysis, four did not utilize an exercise intervention (45, 46, 49, 53), and one solely explored the effects of physical exercise on positive attentional bias (68). After excluding these five studies, the remaining 25 were categorized into high-, moderate-, and low-intensity groups based on the exercise intensity described in the literature. A meta-analysis was then conducted to explore the effects of exercise intensity on negative attentional bias. For high-intensity physical exercise, the results showed substantial heterogeneity (I² = 58.1%, *P* < 0.01), thus, a random-effects model was used. The analysis indicated that high-intensity physical exercise did not significantly reduce negative attentional bias [SMD = −2.05, 95% CI (−3.59, 0.52), *P*> 0.05]. For moderate-intensity physical exercise, heterogeneity was also substantial (I² = 45.1%, *P* < 0.05), therefore, a random-effects model was applied. The results indicated that moderate-intensity physical exercise significantly reduced negative attentional bias [SMD = −1.97, 95% CI (−2.52, −1.41), *P* < 0.01]. For low-intensity physical exercise, heterogeneity was low (I² = 30.1%, *P* = 0.11), thus, a common-effects model was used. The results showed that low-intensity physical exercise did not significantly reduce negative attentional bias [SMD = −0.99, 95% CI (−1.26, 0.72), *P*> 0.05]. The differences between high-, moderate-, and low-intensity physical exercise were marginally significant [SMD = −1.79, 95% CI (−2.23, −1.25), *P* = 0.06]. See **Figure 10**.

#### 3.9.9 Meta-analysis of the effects of different types of physical exercise on negative attentional bias

**Figure 11:**
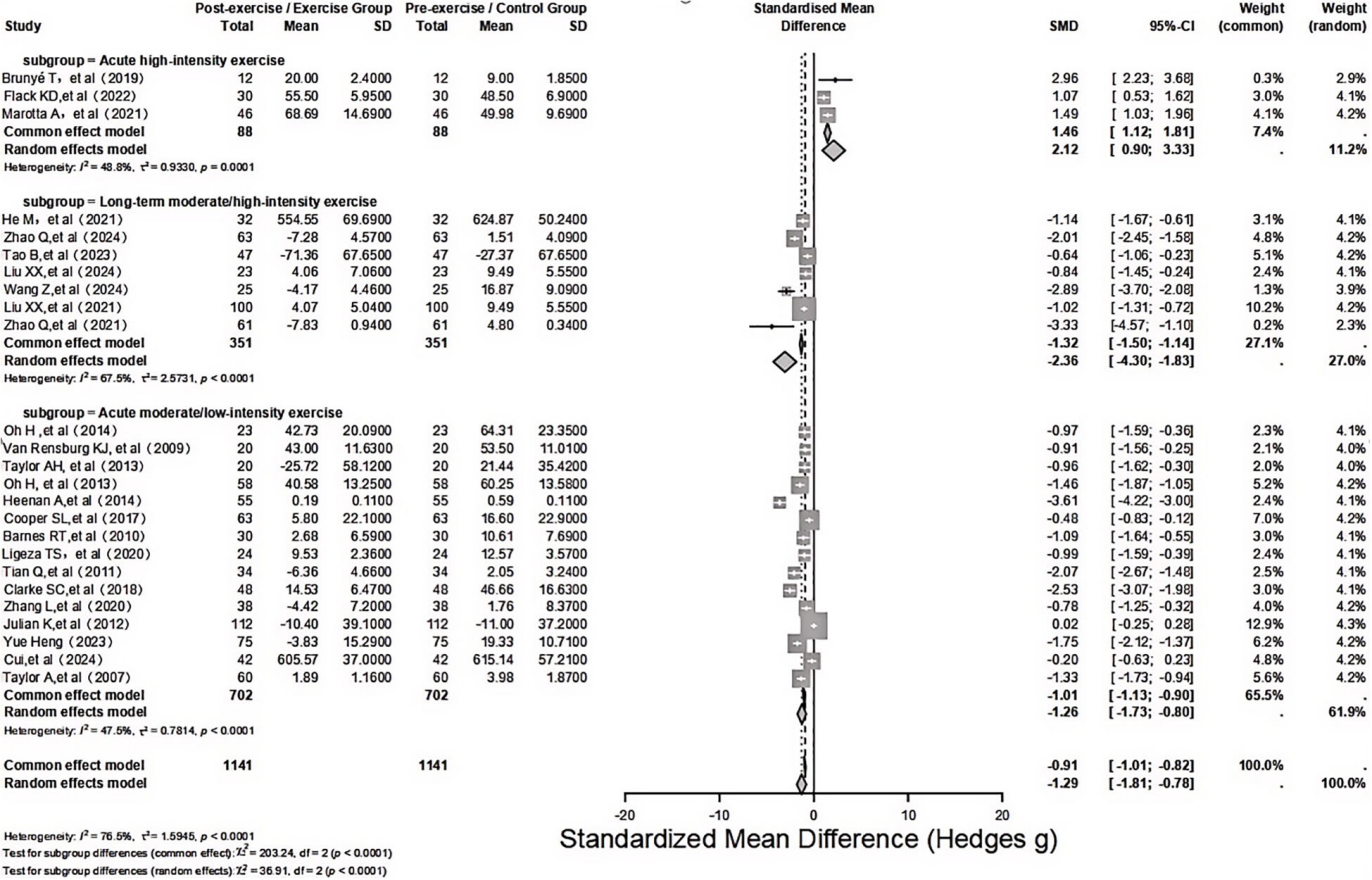
Meta-analysis of the effects of different physical exercise types on individual negative attentional bias.

Based on the exercise types described in the included studies, the remaining 25 studies were further categorized into three groups: acute moderate-/low-intensity exercise, acute high-intensity exercise, and long-term moderate-/high-intensity exercise. A meta-analysis was conducted to examine changes in negative attentional bias across these exercise types. For the acute high-intensity exercise group, heterogeneity was low (I² = 48.8%, *P* < 0.01), therefore, a common effects model was applied. The results indicated that acute high-intensity exercise significantly increased negative attentional bias [SMD = 1.46, 95% CI (1.12, 1.81), *P<* 0.01]. In the long-term moderate-/high-intensity exercise group, heterogeneity was relatively high (I² = 67.5%, *P* < 0.01), thus, a random-effects model was used. The results showed that long-term moderate-/high-intensity exercise significantly reduced negative attentional bias [SMD = −2.36, 95% CI (−4.30, −1.83), *P* < 0.01]. In the acute moderate-/low-intensity exercise group, heterogeneity was low (I² = 47.5%, *P<* 0.01), therefore, a common-effects model was applied. The results showed that this type of exercise significantly reduced negative attentional bias [SMD = -1.01, 95% CI (-1.13, -0.90), *P* < 0.01]. There were significant differences among the acute high-intensity, acute moderate-/low-intensity, and long-term moderate-/high-intensity exercise groups [SMD = −1.29, 95% CI (−1.81, −0.78), *P* < 0.01] (see **Figure 11**).

#### 3.9.10 Sensitivity analysis

A sensitivity analysis of **Figure 5** was conducted using the leave-one-out method. After excluding the study by Flack et al. (52), the pooled effect size was recalculated as SMD = −1.6 [95% CI (−2.9, −1.2), *P* < 0.01], with I² decreasing from 88.0% to 27.7%. The use of high-intensity exercise as the intervention in the excluded study may be the primary source of the high heterogeneity observed in the original meta-analysis. Sensitivity analyses were also performed for the high-intensity exercise group and the between-group comparisons in **Figure 10**. After excluding the study by Zhao et al. (72),, the pooled effect size for the high-intensity exercise group was SMD = −0.78 [95% CI (−1.57, 0.62), *P* > 0.05], with I² decreasing from 58.1% to 30.7%. Upon further excluding the study by Flack et al. (52), I² decreased from 30.7% to 10.1%, indicating a further reduction in heterogeneity. The study by Zhao et al. (72) utilized the attentional bias index as the evaluation metric, while Flack et al. (52) employed high-intensity exercise requiring substantial energy expenditure (500 kcal), these methodological differences may have contributed to the high heterogeneity. After excluding these outliers, heterogeneity decreased (I² from 56.7% to 31.2%), and the differences among high-, moderate-, and low-intensity exercise groups became significant [SMD = −12.6, 95% CI (−18.2, −8.9), *P* < 0.01], with the medium-intensity group showing the most significant effect in reducing negative attentional bias. Further sensitivity analyses were performed for the long-term moderate-/high-intensity exercise group and the between-group comparisons in **Figure 11**. After excluding the study by Zhao et al. (72),, the pooled effect size for the long-term moderate-/high-intensity exercise group was SMD = −1.38 [95% CI (−2.51, −1.02), *P* < 0.01], with I² decreasing from 67.5% to 20.1%. Similarly, after excluding this study, between-group heterogeneity decreased (I² from 76.5% to 48.1%), and significant differences were observed among the acute high-intensity, long-term moderate-/high-intensity, and acute moderate-/low-intensity exercise groups [SMD = −1.11, 95% CI (−2.21, −0.91), *P* < 0.01].

#### 3.9.11 Publication bias assessment

**Figure 12:**
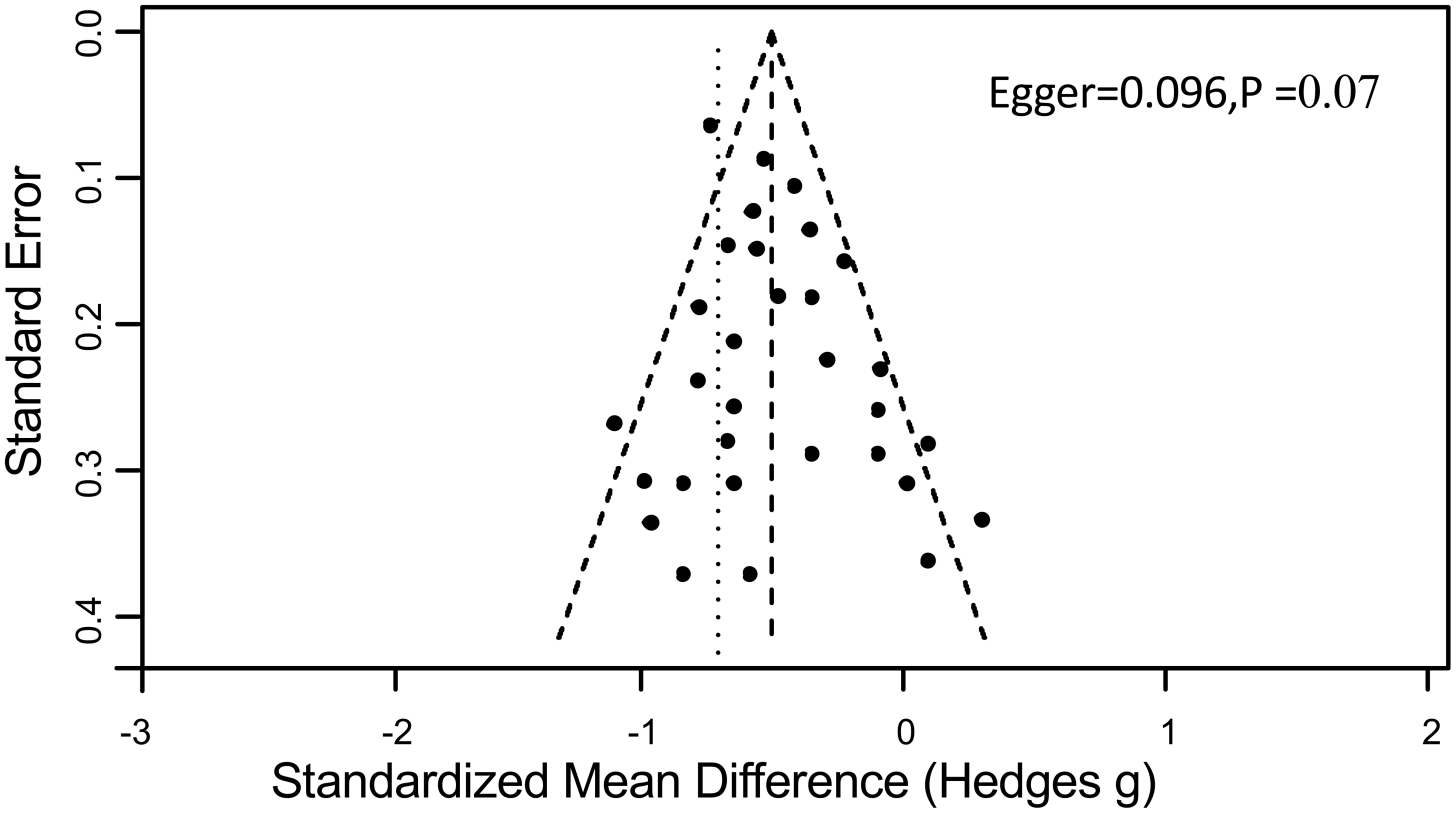
Funnel plot of the included studies related to negative attentional bias.

Note: P value represents the significance level of Egger’s test; *P* > 0.05 indicates no risk of publication bias.

Publication bias was assessed for the outcome of negative attentional bias, which had the largest number of included studies. The risk of publication bias was evaluated using funnel plots and Egger’s test (75). The funnel plot displayed an approximately symmetrical distribution, and Egger’s test yielded a P-value of 0.096, indicating no significant risk of publication bias among the included studies. See **Figure 12**.

## 4 Discussion

The aim of this study was to systematically review and analyze the relationship between physical exercise and attentional bias through a meta-analysis, providing a comprehensive understanding of the current state of research in this field. A total of 32 studies were included in this review, involving target populations such as individuals with anxiety and depression, addiction, overweight or obesity, and those experiencing high stress. The focus of these studies can be categorized into two main aspects: the effects of physical exercise on negative and positive attentional bias. The results of the review and meta-analysis indicate that physical exercise may, to some extent, reduce negative attentional bias and increase attentional bias towards positive information.

### 4.1 The relationship between physical exercise and individual negative attentional bias

Negative attentional bias is closely associated with emotional disorders, maladaptive behaviors (sedentary lifestyle, addiction) and substance dependence, and similar conditions, thus garnering significant scholarly attention. Among the 32 included studies, 26 explored the relationship between physical exercise and negative attentional/interpretational bias. Among these, there were ten studies involving individuals with addiction (32, 33, 57, 58, 61–63, 69, 72, 73), four studies involving individuals with anxiety and depression (44, 51, 55, 67), four involving individuals with obesity or overweight (52, 60, 65, 71), six involving healthy individuals (47, 50, 54, 56, 59, 66), and two involving high-stress college students (64, 74). The findings of these 26 studies were generally consistent, indicating that physical exercise reduces negative attentional/interpretational bias and increases positive attentional bias. However, Cooper et al. (51) and Julian et al. (55) reported inconsistent findings, utilizing textual stimuli, they observed no significant effect of physical exercise on reducing negative attentional bias, possibly because textual stimuli require more abstract information processing such as semantic coding, cognitive engagement, or threat assessment. The effect of physical exercise on thus complex cognitive processes may be limited. In addition, Brunyé et al. (47), Flack et al. (52), and Marotta et al. (59) found that physical exercise increased attentional bias toward negative cues (**Figure 11**), a result inconsistent with **Figure 10**, where high-intensity exercise showed no significant effect. This discrepancy may be related to exercise load. In the analysis for **Figure 11**, Flack et al. (52) utilized an acute session expending 500 kcal, Marotta et al. (59) used a half marathon (21km, duration >1h), and Brunyé et al. (47) employed a high-intensity protocol (75-85% of peak HR). Overall, these three studies employed a single bout of high-intensity acute exercise, whereas other studies used either acute moderate-intensity or long-term moderate-to-high intensity exercise. For example, although seven studies in **Figure 10** used high-intensity exercise, those protocols were periodic and included recovery components. Differences in exercise intensity and modality might account for these inconsistent findings. From a physiological perspective, a single bout of acute high-intensity exercise can act as a stressor, particularly due to increased energy consumption, elevated body temperature, lactic acid accumulation, muscle soreness, and shortness of breath. These factors can increase negative emotional experiences (76), and negative emotions are closely related to negative attentional bias (10). According to mood-congruency theory, individuals tend to process information consistent with their current mood first, while being less sensitive to incongruent information. The increased negative attentional bias observed by Brunyé et al. (47),, Flack et al. (52) and Marotta et al. (59) may be related to the negative mood induced by high-intensity exercise. These findings suggest that high-intensity or high-energy-expenditure exercise per se may be less effective in improving attentional bias, whereas moderate-intensity or short-duration exercise may be more beneficial. Apart from these three contradictory studies (47, 52, 59), 20 studies support the view that physical exercise reduces negative attentional bias (32, 33, 44, 50, 54, 56–58, 60–67, 69, 71–73). Although Cooper et al. (51) found no significant effect using textual materials, they observed a reduction in negative attentional bias when using image-based materials with anxious individuals. Similarly, although Zhao et al. (72) employed high-intensity exercise (80%–85% HRmax), they found it reduced negative attentional bias. Therefore, it can be inferred that using image-based materials and moderate-intensity exercise as interventions can effectively reduce negative attentional bias. Conversely, when word-based materials were used, the effect was not obvious. This may be because visual information possesses higher emotional valence and ecological validity, making it more easily detected by the sensory system. This is consistent with the threat appraisal model in cognitive psychology (77), which suggests that individuals are more sensitive to high-threat or high-arousal information. The effects of physical exercise on attentional bias appear subject to specific boundary conditions. Moderate-intensity exercise may reduce negative bias, whereas high-intensity exercise, word-based stimuli, or specific populations (e.g., obese individuals undergoing high energy-expenditure) may show no significant effects or even adverse effects. This inference is supported by the high heterogeneity observed in **Figures 5** and **10**. The meta-analysis in **Figure 5** showed substantial heterogeneity among three studies (52, 60, 71) investigating overweight or obese individuals. Given that this subgroup included only three studies, the interpretation of this heterogeneity should be considered preliminary and speculative. However, the leave-one-out sensitivity analysis provided valuable insight: after excluding Flack et al. (52), heterogeneity decreased substantially. Flack et al. (52) employed high energy-expenditure exercise (500 kcal), whereas Oh and Taylor (60) and Zhang et al. (71) used moderate-intensity, short-duration interventions. Based on this observation within the specific subgroup, we cautiously speculate that differences in energy expenditure may be a potential source of this heterogeneity. It is possible that high-intensity or excessive energy-expenditure exercise, acting as a potent stressor, may exacerbate negative attentional bias, while moderate-intensity exercise facilitates the regulation of physiological and psychological states. It must be explicitly acknowledged that, due to the limited sample size (n=3), these inferences regarding the source of heterogeneity are exploratory and require verification in future studies with larger samples. Furthermore, valuable studies by Barnes et al. (44), Schmitt et al. (78), and Thom et al. (79), from attention control and neurophysiological perspective, respectively, confirmed that physical exercise reduces negative attentional bias. Some studies suggested that exercise enhances “top-down” cognitive control, thereby suppressing the “bottom-up” influence of emotional distractors and reducing attentional bias toward threatening stimuli (37). However, findings by Schmitt et al. (78), Thom et al. (79), and Yu et al. (37) require further validation. Cognitive psychology studies confirmed that attention dissociation difficulties from negative stimuli is a key cause of negative attentional bias, that is, the inability to shift attention away results in sustained attention to negative information. A previous study demonstrated (67) that moderate-intensity exercise reduced attention dissociation difficulties in anxious individuals, suggesting that exercise accelerates attention dissociation from negative stimuli. This mechanism may underlie the reduction in negative attentional bias following exercise. Additionally, physical exercise enhances attentional control, enabling flexible shifting of attention across stimuli, and further reducing negative attentional bias (80).

### 4.2 The relationship between physical exercise and individual positive attentional bias

While most scholars have focused on the relationship between physical exercise and negative attentional bias, eight studies in this review explored the effect of physical exercise on positive attentional bias (45, 46, 48, 49, 53, 68, 70, 74). These studies involved depressed individuals (68), anorexia patients, athletes and non-athletes (53), community residents (70), and college students (45, 46, 48, 49, 74). In addition to differences in participants, the experimental materials varied. Yang et al. (68) used emotional image pairs, and Cui et al. (74) used emotional face pairs, while the remaining six studies utilized images or word pairs related to physical exercise (45, 46, 48, 49, 53, 70). Given that physical exercise is beneficial to both physical and mental health, such images or words can be regarded as positive cues. Despite these differences, the results of the intervention studies were consistent in suggesting that physical exercise enhances positive attentional bias. Yang et al. (68) observed that improvement in positive attentional bias among depressed individuals were accompanied by a reduction in depressive symptoms. Yun et al. (70) found that attentional bias toward physical exercise-related images could significantly predict levels of leisure-time physical activity. Cui et al. (74) asked 42 college students with high psychological stress to complete a 30-minute basketball session (60–70% of maximal oxygen uptake). The results showed that the participants’ reaction time to positive emotional words increased following the exercise session. Thus, improving positive attentional bias appears to be an important factor in ameliorating emotional disorders, correcting adverse behaviors (such as sedentary lifestyle and addiction), and promoting healthy behaviors (such as engagement in physical exercise or activity). It should be noted that six of the aforementioned studies (45, 46, 48, 49, 53, 70) did not involve exercise interventions but instead explored the relationship between attentional bias toward exercise-related information and exercise behavior based on cross-sectional surveys. Strictly speaking, these six studies differ slightly from the primary focus of the present study. However, they confirm from another perspective that enhancing attentional bias toward exercise (positive attentional bias) is a potential measure to promote individual exercise behavior (positive behavior). Amir et al. (20) and Field et al. (81) studied individuals with anxiety and alcohol addiction, respectively, confirming the significant role of increasing positive attentional bias in improving adverse psychological states and substance addiction outcomes. The mechanism by which physical activity enhances positive attentional bias can be explained by the attention control theory proposed by Barnes et al. (44). Furthermore, Schmitt et al. (78) used fMRI to confirm that post-exercise increases in positive attentional bias and decreases in negative attentional bias were related to changes in emotional processing patterns induced by fearful faces in the brain. Specifically, physical activity reduced activation in the right and part of the left caudate nucleus, which in turn reduced the responsiveness of neurons in these regions to fearful faces. Babyak et al. (82) also reported that physical exercise enhances the processing of positive emotional cues by increasing dopamine (DA) and serotonin (5-HT) levels in relevant brain regions. Although these valuable studies provide strong support for the findings of this review, they did not meet the inclusion criteria for the meta-analysis and were therefore excluded. Nevertheless, their contributions to the field remain noteworthy (20, 77, 82, 83). In summary, investigating the improvement of individual positive attentional bias through physical exercise provides a new theoretical perspective for further elucidating the mechanism by which exercise improves mental, behavioral and physical health outcomes.

### 4.3 Limitations of the study and suggestions for future research

This article systematically reviews the relationship between physical exercise and attentional bias (both positive and negative), providing a basis for comprehensively understanding the current state of research in this field. This study also has certain implications for the elucidating the mechanisms underlying mental and behavioral disorders in individuals with anxiety, depression, obesity, overweight, addiction, and high psychological stress, as well as for formulating physical exercise intervention programs. However, several limitations should be acknowledged. The primary limitations stem from the wide range of exercise types included, ranging from organized and purposeful training to daily physical activity. Heterogeneity in exercise types, experimental paradigms (e.g., dot-probe task, Stroop task, eye-tracking methods), participant characteristics (e.g., addiction, anxiety/depression, overweight/obesity, high stress, healthy individuals), and assessment tools hampers the direct comparison of results across studies. Furthermore, while some researchers observed the relationship between physical activity and negative attentional bias, others examined the effects of physical exercise intervention. Given the contradictory findings reported in some studies, the results of this review should be interpreted with caution. Future research should adopt more standardized experimental paradigms to conduct comparative studies on negative attentional bias following exercise intervention across different groups, in order to identify individuals who are more responsive to such interventions. Additionally, comparing changes in attentional bias under different exercise forms, intensities and durations is crucial for determining optimal intervention protocols and represents a key direction for future research. Moreover, it is important to recognize that some subgroup analyses included only a limited number of studies (e.g., only three studies in the overweight/obesity subgroup), which precludes robust conclusions regarding the sources of heterogeneity and may increase the risk of publication bias. Additionally, the meta-analysis involved statistical imputations for studies lacking pre-intervention data. Specifically, a zero-baseline imputation (mean=0, SD = 0) was applied, which mathematically equates the change score analysis to a post-test comparison for these studies. Although sensitivity analyses and ICC robustness checks indicated that the pooled effects were not significantly distorted, this approach assumes baseline equivalence between groups and fails to account for true baseline variance. Consequently, these specific findings and heterogeneity interpretations should be interpreted with caution. Future original studies investigating the effects of exercise on attentional bias in overweight or obese individuals would help further verify the robustness of our findings. Finally, the studies included in this review were conducted in both Eastern and Western cultural contexts. Such cultural differences may potentially influence attentional bias. For instance, Western culture tends to encourage direct emotional expression, whereas Eastern culture often prefers indirect and subtle modes of expression. Similarly, while red symbolizes luck, prosperity, and celebration in Chinese culture, it is commonly associated with blood, aggression, provocation, or unrest in Western perceptions. Future research should explore the potential moderating effects of cultural contexts on attentional bias.

## 5 Conclusion

Despite a few inconsistent findings, the majority of studies support the conclusion that physical exercise reduces negative attentional bias and increases positive attentional bias across various populations. Notably, moderate-intensity exercise generally demonstrates more positive effects. The results indicate that moderate-intensity exercise reduces negative attentional bias and increases positive attentional bias in individuals with high psychological stress, anxiety, depression, addiction, and healthy individuals, although the effect on negative attentional bias in overweight or obese individuals appears limited. However, the validity of these conclusions is contingent upon specific conditions (1): exercise intensity is moderate (typically 40%-70% HRmax or RPE 11-13) (2); assessment tools possess high ecological validity (e.g., image stimuli rather than text stimuli); and (3) the intervention does not involve a single session of high-energy-expenditure exercise (>500 kcal) or extremely long-duration exercise (>1 hour). Overall, these findings offer a new perspective for understanding the potential mechanisms by which physical activity improves mental disorders (e.g., anxiety, depression) and maladaptive behaviors (e.g., sedentary lifestyle, behavioral addiction, substance addiction). This study has important clinical implications. Future research should focus on developing integrated intervention protocols that combine moderate-intensity aerobic exercise with attentional bias modification (ABM) training for individuals with anxiety and substance dependence.

## Data Availability

The raw data supporting the conclusions of this article will be made available by the authors, without undue reservation.

## Appendix

All questions are answered with ‘yes’ or ‘no’.

Section 1: Study design

(1)Was the research question/objective clearly stated?

(2)Were all subjects selected or recruited from the same or similar populations (including the same time period)?

(3)Were the inclusion and exclusion criteria for being in the study pre-specified and applied uniformly to all participants?

(4)Was the study population and size clearly specified and defined?

Section 2: Data collection and methodology

(1)Were standardized measures used, or where measures are designed for the study, attempts to ensure reliability and validity were made?

(2)Were the data collected in a way that addressed the research issue?

(3)Was the participation rate stated and at least 50%?

(4)Was the number of participants described at each stage of the study?

(5)If the study followed participants up, were reasons for loss to follow-up explained?

Section 3: Analysis and interpretation of results

(1)Were details of statistical tests and confidence intervals sufficiently rigorous and described?

(2)Were potential confounding variables measured and adjusted statistically for their impact on the relationship between exposure(s) and outcome(s)?

(3)Was the answer to the study question provided?

(4)Are the findings related back to previous research?

(5)Do conclusions follow from the data reported?

(6)Are conclusions accompanied by the appropriate caveats?
